# A Reduction in Age-Enhanced Gluconeogenesis Extends Lifespan

**DOI:** 10.1371/journal.pone.0054011

**Published:** 2013-01-14

**Authors:** Mayumi Hachinohe, Midori Yamane, Daiki Akazawa, Kazuhiro Ohsawa, Mayumi Ohno, Yuzu Terashita, Hiroshi Masumoto

**Affiliations:** 1 National Food Research Institute, National Agriculture and Food Research Organization (NARO), Tsukuba, Ibaraki, Japan; 2 Faculty of Life and Environmental Sciences, University of Tsukuba, Tsukuba, Ibaraki, Japan; University of Minnesota, United States of America

## Abstract

The regulation of energy metabolism, such as calorie restriction (CR), is a major determinant of cellular longevity. Although augmented gluconeogenesis is known to occur in aged yeast cells, the role of enhanced gluconeogenesis in aged cells remains undefined. Here, we show that age-enhanced gluconeogenesis is suppressed by the deletion of the *tdh2* gene, which encodes glyceraldehyde-3-phosphate dehydrogenase (GAPDH), a protein that is involved in both glycolysis and gluconeogenesis in yeast cells. The deletion of *TDH2* restores the chronological lifespan of cells with deletions of both the *HST3* and *HST4* genes, which encode yeast sirtuins, and represses the activation of gluconeogenesis. Furthermore, the *tdh2* gene deletion can extend the replicative lifespan in a CR pathway-dependent manner. These findings demonstrate that the repression of enhanced gluconeogenesis effectively extends the cellular lifespan.

## Introduction

Aging is a complex process that is associated with the gradual loss of physiological functions, which are regulated by genetic and environmental factors. Recent studies in genetically tractable model systems including yeast, worms, flies and mice demonstrate that longevity can be modulated by single gene mutations [1], [2], [3], [4], [5], [6]. Calorie restriction (CR) is the most effective intervention known to extend lifespan in a variety of metazoan species [7], [8]. CR has also been shown to delay the onset or reduce the incidence of many age-related diseases, including cancer, diabetes and cardiovascular disorders [8], [9], [10]. CR may work by reducing the level of reactive oxygen species (ROS) as a result of the slowed energy metabolism [7], [8]. Although the mechanism by which CR extends longevity and ameliorates age-associated diseases remains unclear, energy metabolism has a key role in longevity via the CR pathway. The central carbon metabolism pathway, which includes the glycolysis/gluconeogenesis and tricarboxylic acid (TCA) pathways, uses various carbon sources, such as glucose and glycerol, to produce ATP molecules. The accumulation of metabolic intermediates produced by enhanced gluconeogenesis has been reported to be an age-induced change in budding yeast [11]. However, the role of augmented gluconeogenesis in cellular aging remains unclear.

Sir2 family proteins (sirtuins) are evolutionally conserved and were originally discovered and studied in yeast as a component of the Sir1/2/3/4 silencing complex [12], [13]. The mammalian orthologs of *SIR2* encode nicotinamide adenine dinucleotide (NAD^+^)-dependent protein deacetylases and ADP-ribosylases [14]. Previous studies have shown that the sirtuins play important roles in cellular longevity in yeast and in regulating the stress response, cell survival and energy metabolism in multicellular organisms [15]. Earlier studies showed that sirtuins play important roles in CR-induced lifespan extension [16], [17] and in regulating the stress response, cell survival and energy metabolism, suggesting a role for sirtuins in age-related metabolic diseases [15], [18], [19]. The Sir2 homologs in yeast include Hst1, Hst2, Hst3 and Hst4 [20]. Of these, Hst1 exhibits the highest homology with Sir2 and mediates transcriptional regulation independent of the *SIR* silencing complex [20]. Hst2 functions in concert with Hst1 to downregulate subtelomeric gene expression [21] and plays a role in regulating rDNA silencing and recombination [22], [23]. Hst3 and Hst4 together maintain telomeric silencing and cell cycle progression [20]. Hst3 and Hst4 deacetylate histone H3 on lysine 56 (H3-K56) in chromatin during S phase to the next G1 phase to prevent the genomic instability caused by the continuous acetylation of H3-K56 [24].

Recent studies have shown that several longevity factors and pathways are highly conserved among eukaryotes [3]. The budding yeast *S. cerevisiae* provides an efficient model for exploring the molecular mechanism of longevity regulation. Budding yeast propagate by asymmetric cell division, in which the partitioning between the two resulting cells is unequal morphologically and molecularly. The larger cell is designated as the mother cell, and each yeast cell can only undergo a certain number of cell divisions, known as the replicative life span (RLS). The process of approaching the limit of cell divisions is recognized as replicative aging. Similar to higher eukaryotic cells, aged yeast cells exhibit a declining division potential and reduced fitness. Another type of yeast lifespan is chronological lifespan (CLS), which measures the length of time that cells remain viable in a non-dividing state. Yeast cells enter the non-dividing stationary phase (or post-diauxic phase) when nutrients are limited. This quiescent state has been suggested to resemble the G0 state in higher eukaryotes [25]. Several longevity factors have been identified through RLS and CLS studies [26], [27], [28], [29], [30]. The yeast rDNA loci consist of a stretch of approximately 9 kb rDNA repeats. Homologous recombination between adjacent repeats is known to result in the excision of repeat units and the formation of extrachromosomal rDNA circles known as ERCs. ERCs are autonomously replicating and preferentially accumulate in the aging mother cell [31]. Sir2 counteracts ERC formation via the establishment and/or maintenance of sister-chromatid cohesion at the rDNA loci [32]. Sir2 also plays a role in stress resistance and the regulation of newborn cell fitness by conferring a superior ROS management to the daughter cell to protect the progeny from aging [33]. Similar to cells harboring deficiencies in DNA damage repair, cells with deletions of both the *HST3* and *HST4* genes display short RLSs due to the genome instability that accumulates with each cell division [34], [35], [36]. Interestingly, *hst3*Δ cells reduce CLS [37]. It is unclear why the quiescent *hst3*Δ cell exhibits a reduced chronological lifespan under nutrient starvation.

In this study, we determined that the short CLS of *hst3*Δ *hst4*Δ cells was due to age-enhanced gluconeogenesis. We isolated several genes encoding metabolic enzymes to restore the CLS of *hst3*Δ *hst4*Δ cells. Among these isolated genes, the deletion of the *TDH2* gene, which encodes an isoform of glyceraldehyde-3-phosphate dehydrogenase (GAPDH), which is essential for glycolysis/gluconeogenesis, reduced the amount of accumulated metabolites from glucose metabolism in *hst3*Δ *hst4*Δ cells and specifically repressed gluconeogenesis. Furthermore, the RLS was extended in cells in which the *TDH2* gene was deleted in a manner similar to that induced by the CR pathway. Our data suggest that Hst3 and Hst4 coordinately regulate age-enhanced gluconeogenesis to maintain the CLS, and that a reduction in age-enhanced gluconeogenesis can extend the cellular lifespan.

## Materials and Methods

### Replicative Lifespan (RLS) Assay (Pedigree Analysis)

The RLS assay, known as pedigree analysis, was performed as described previously [38]. Typically, a minimum of 50 mother cells was counted for each strain tested. To compare the difference of replicative lifespans between strains on statistics, we performed the Wilcoxon Rank-Sum test.

### Chronological Lifespan (CLS) Assay

CLS assays were conducted in 0.5% glucose synthetic complete (SC) media [39] supplemented with a fourfold excess of the amino acids for which the strains were autotrophic in the absence (“unbuffered”) of 50 mM citrate-phosphate buffer [pH6.0]. Yeast cells were cultured overnight in YPD at 25°C. The cells (5×10^6^ cells/ml) were suspended in SC medium (0.5% glucose) and cultured at 30°C. Cell viability was determined every 2 days. To determine viability, the cells were washed with 500 µl of PBS pH 7.3 and mixed with 500 µl of 15 µg/ml phloxin B solution [40]. Cell viability was calculated from the number of red-stained cells among the total cell number (n = 100). Almost all BY4742 cells were stained with phloxin B at the start in the presence of 2% glucose, but not in 0.5% glucose. Therefore, we used SC medium containing 0.5% glucose to perform the CLS assay by phloxin B staining.

### Growth Rate Assay in Liquid Culture

After being cultured overnight in YPD at 25°C, the cells (5×10^6^ cells/ml) were suspended in SC medium (0.5% glucose) and cultured at 25°C without shaking. The cell number was determined every 24 h using a Z-1 Coulter Counter (Beckman-Coulter Co., IN. USA). At least three replicates were analyzed for each strain.

### Preparation of Yeast Cell Extract

The cell extract was prepared according to a previously described method [11], [41]. Briefly, mid-log growth yeast cells (1×10^8^ cells in total) cultured in YPD were harvested and washed three times with PBS buffer (pH 7.3). The cells were suspended in 500 µl of metabolic enzyme extraction buffer (20 mM sodium phosphate pH 7.5, 0.02% bovine serum albumin [BSA], 0.5 mM ethylenediaminetetraacetic acid [EDTA], 5 mM ß-mercaptoethanol, 25% glycerol, 0.5% Triton X-100). The cell suspension was mixed with same volume of acid-washed glass beads (diameter of less than 0.5 mm), and the cells were disrupted by vortexing. Soluble proteins were obtained by collecting the supernatant after centrifugation of the broken cell suspension at 14,000 rpm for 5 min at 4°C. The protein concentration was determined by the Bradford protein assay with BSA as the standard.

### GAPDH Glycolytic Assay

The GAPDH glycolytic assay was based on a previously described method [42]. The assay was conducted at 30°C by adding the cell extract (40 µg) to a substrate solution containing 100 mM Tris/HCl (pH 8.5), 1.5 mM NAD^+^, 5.0 mM sodium phosphate (pH 7.0) and 3.2 mM D-glyceraldehyde-3-phosphate. Changes in absorbance (340 nm) were monitored spectrophotometrically (DU 800, Beckman Coulter Co.).

### GAPDH Gluconeogenic Assay

The gluconeogenic substrate of GAPDH, 1,3-diphosphoglycerate, was generated by the following previously described method [42]. The reaction mixture was composed of 80 mM triethanolamine (pH 8.5), 8.0 mM MgSO_4_, 0.25 mM NADH, 2.4 mM ATP, 12 mM 3-phosphoglycerate and 18.8 µg/ml budding yeast phosphoglycerol kinase (Sigma-Aldrich, USA). The reaction was conducted at 37°C for 30 min and stopped by heating at 100°C for 5 min. The assay was performed at 30°C by mixing the cell extract (4 µg), 0.05 mM NADH and 200 µl of the reaction mixture containing 1,3-diphosphoglycerate. The changes in absorbance (340 nm) were monitored with a spectrophotometer.

### Glycogen Quantification Assay

The cell pellet harboring glycogen was prepared according to the following previously described method [43]. The cells were cultured at 30°C (25°C was used for *hst3*Δ *hst4*Δ and *tdh2*Δ *hst3*Δ *hst4*Δ cells) for 3 h in either YPD or YPE liquid media. After harvesting and washing three times with PBS buffer (pH 7.3), the cells (1×10^8^) were suspended in 300 µl of 30% KOH and incubated at 100°C for 2 h. The cell suspension was mixed with 2 volumes of 99% ethanol and centrifuged at 14000 rpm for 5 min at 4°C. The cell pellet was dried and suspended in 100 µl of H_2_O. The cell suspension (10 µl) was assayed with a Glycogen Assay Kit (Biovision Co., USA) according to the manufacturer’s instructions. The changes in absorbance (570 nm) were monitored with a microplate reader (iMark, BioRad Co., USA).

### Reactive Oxygen Species (ROS) Detection

The protocol was modified as previously described [44]. The cells (5×10^7^) were washed with PBS (pH 7.3) and resuspended in 100 µl of PBS containing 5 µg/ml dihydroethidium (DHE). After incubation at room temperature for 5 min, the cells were harvested and suspended in 100 µl of PBS (pH 7.3). DHE-positive cells were viewed under rhodamine fluorescence using a Leica CTR6500 microscope with LAS AF software. The ratio was calculated from the number of DHE-positive cells among the total number of cells (n = 100).

## Results

### hst3Δ hst4Δ Cells Exhibit Reduced Chronological Lifespan in a Manner Independent of the Regulation of Histone H3-K56 Acetylation

**Figure 1:**
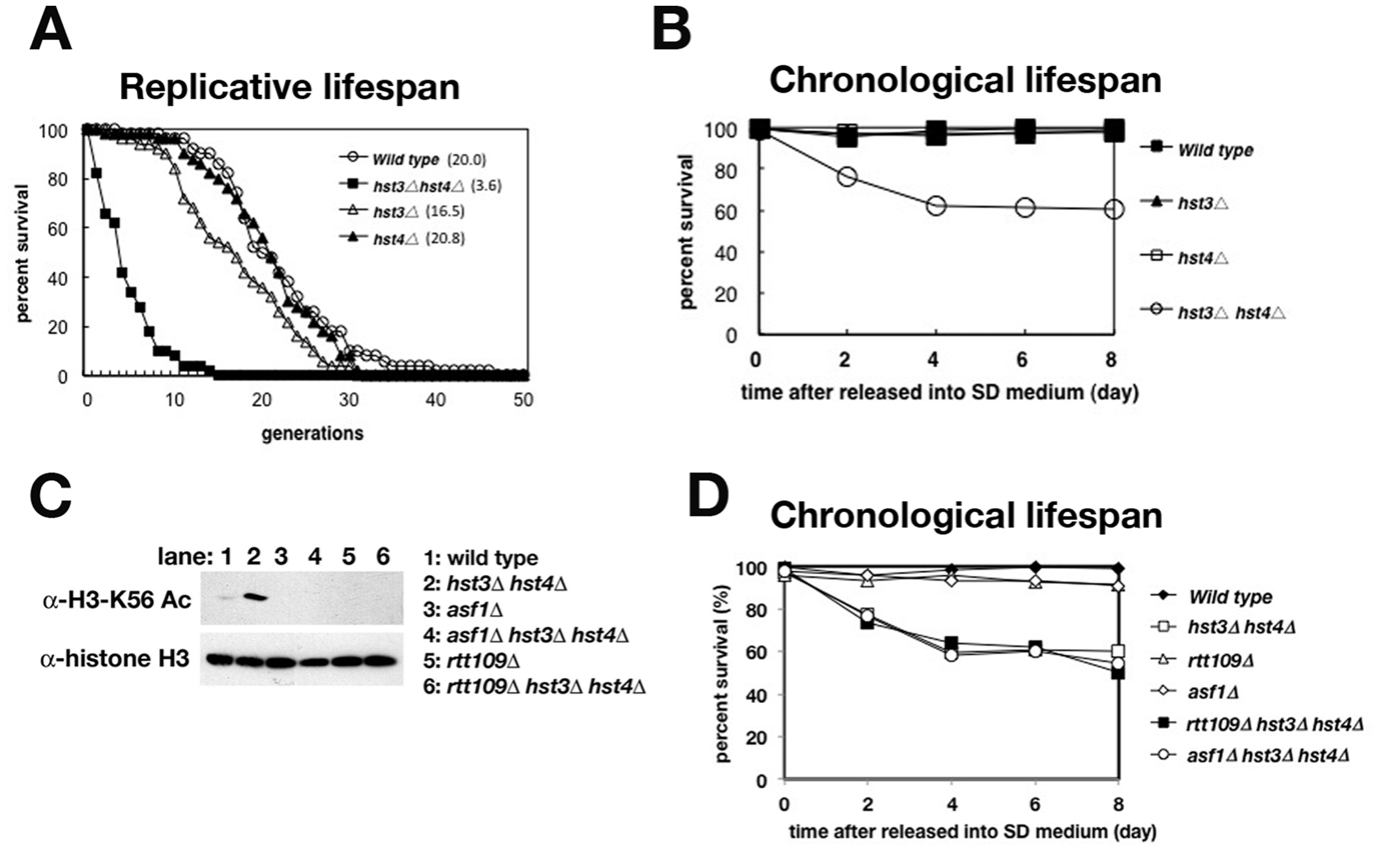
*hst3*Δ *hst4*Δ cells reduce CLS independent of the continuous acetylation of histone H3-K56. (A) Replicative lifespan (RLS) of wild type (BY4742), *hst3*Δ, *hst4*Δ and *hst3*Δ *hst4*Δ cells. The median lifespan is given next to each genotype. The difference between strains was performed on statistical calculations (Fig. S1) (B) Chronological lifespan (CLS) analysis of wild type, *hst3*Δ, *hst4*Δ and *hst3*Δ *hst4*Δ cells. (C) Immunoblot analysis of whole-cell protein extracts using antibodies either against acetylation of histone H3 on K56 or total histone H3 as a loading control. Cell extracts were prepared from asynchronized wild type, *asf1*Δ, *rtt109*Δ, *hst3*Δ *hst4*Δ, *asf1*Δ *hst3*Δ *hst4*Δ and *rtt109*Δ *hst3*Δ *hst4*Δ cells. Each cell extract was resolved by 15% SDS-polyacrylamide gel electrophoresis (PAGE) and analyzed by immunoblot using antibodies to detect K56-acetylated H3, and total histone H3. (D) CLS analysis of wild type, *hst3*Δ *hst4*Δ, *rtt109*Δ, *asf1*Δ, *rtt109*Δ *hst3*Δ *hst4*Δ and *asf1*Δ *hst3*Δ *hst4*Δ cells.

As has been described previously [34], [35], [36], *hst3*Δ *hst4*Δ cells exhibited a short RLS (Figs. 1A and S1). Interestingly, *hst3*Δ *hst4*Δ cells also exhibited a short CLS following the accumulation of reactive oxygen species (ROS) (Figs. 1B and S2). Recent research shows that pH of the medium affects the cell viability in the CLS assay [45], and we routinely employed SD medium in the absence (“unbuffered”) of 50 mM citrate-phosphate buffer [pH6.0]. To exclude the possibility that the pH alteration would cause to reduce the CLSs of *hst3*Δ *hst4*Δ cells, we confirmed that *hst3*Δ *hst4*Δ cells reduced the viability even in CLS assay using SC medium even in the presence (“buffered”) of 50 mM citrate-phosphate buffer [pH6.0] (Fig. S3). The short RLS of *hst3*Δ *hst4*Δ cells is due to the persistence of H3-K56 acetylation in chromatin that can trigger the genomic instability [24], [36]. Next, we examined whether the short CLS of the *hst3*Δ *hst4*Δ cells also depended on histone H3-K56 acetylation. Rtt109 is a histone acetyltransferase that acetylates histone H3-K56 [46], [47], [48], and Asf1 is a histone chaperone that is necessary for the acetylation of K56 of histone H3 [49], [50]. We confirmed that the acetylation of histone H3-K56 was not detected in *asf1*Δ, *rtt109*Δ, *asf1*Δ *hst3*Δ *hst4*Δ and *rtt109*Δ *hst3*Δ *hst4*Δ cells (Fig. 1C). Cells containing deletions of either *RTT109* or *ASF1* did not exhibit reduced CLS (Fig. 1D). However, both *asf1*Δ *hst3*Δ *hst4*Δ and *rtt109*Δ *hst3*Δ *hst4*Δ triple deletion mutants exhibited a reduced CLS identical to that of the *hst3*Δ *hst4*Δ double deletion mutant (Fig. 1D). Thus, these data suggest that the short CLS of *hst3*Δ *hst4*Δ cells is not due to histone H3-K56 acetylation.

### Metabolic Intermediates from Glucose Metabolism Accumulate in *hst3*Δ *hst4*Δ Cells in a Pattern that is Similar to that of Aged Cell

**Figure 2:**
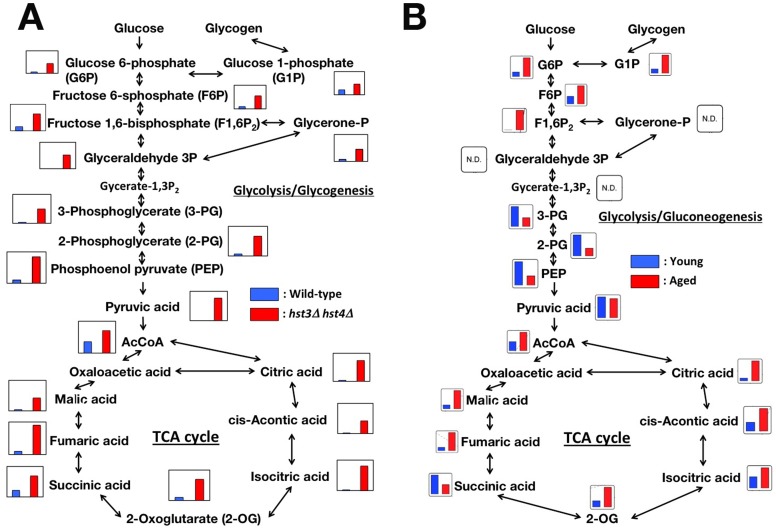
*hst3*Δ *hst4*Δ cells accumulate a number of metabolic intermediates from glucose metabolism in a pattern similar to that of aged cells. (A) The metabolic profiling data of glucose metabolites in wild type and *hst3*Δ *hst4*Δ cells were mapped onto known pathways. Each graph was derived from the calculated amount of metabolic intermediates shown in Table S1. The cells were cultured in SC medium at 30°C for 3 days and analyzed by CE-TOFMS. (B) The metabolic profiling data of central carbon metabolism in both young and aged cells was mapped as in (A). Each graph was derived from the calculated amount of metabolic intermediates shown in Table S2.

The CLS of yeast cells is monitored as cellular viability under the starvation of nutrient, especially glucose. This observation prompted us to hypothesize that the glucose metabolism might not respond to nutrient starvation, and lead to reduce CLSs of the *hst3*Δ *hst4*Δ cells. To evaluate this hypothesis, we analyzed the profiles of the metabolites of glucose metabolism in wild type and *hst3*Δ *hst4*Δ cells using capillary electrospray ionization time-of-flight mass spectrometry (CE-TOFMS) [51]. Wild type and *hst3*Δ *hst4*Δ cells were cultured under the same conditions employed in the CLS assay. The metabolites were prepared from these cells, and analyzed by CE-TOFMS. All intermediates of glycolysis/gluconeogenesis and the TCA cycle that measured in this study accumulated in the *hst3*Δ *hst4*Δ cells more than in the wild type cells (Fig. 2A and Table S1). Several metabolites connecting with glucose storage: glucose-1-phosphate (G1P), glucose-6-phosphate (G6P) and fructose-6-phosphate (F6P), were accumulated in the *hst3*Δ *hst4*Δ cells (Fig. 2A). The accumulation of these metabolites is also observed in rapidly aging *sip2*Δ and aged wild type cells [11]. To confirm the profiles of metabolites in *hst3*Δ *hst4*Δ cells are reminiscent of those of aged cells, we analyzed the profiles of the major energy metabolites in young and aged cells using CE-TOFMS. Aged cells (median 16–20 cell divisions) and young cells (zero or one cell division) were isolated using biotin-streptavidin magnetic sorting [31], [52]. Similar to the metabolic profile of *hst3*Δ *hst4*Δ cells (Fig. 2A), the metabolites involved in glucose storage (G1P, G6P and F6P) and many metabolites of the TCA cycle accumulated in aged cells (Fig. 2B). The pronounced enhancement of glucose storage in aged cells is brought by the augment gluconeogenesis and the reduced glycolysis [11]. The accumulation of the metabolites in the TCA cycle supports that the gluconeogenesis pathway was enhanced, because these metabolites can be utilized to synthesize glycogen via the gluconeogenesis pathway (Figure 4C). Other metabolic processes, such as nucleotide synthesis and amino acid synthesis, did not exhibit global differences in the accumulation of metabolic intermediates in aged and young cells (Fig. S4 and Table S2). Together, these data suggest that the glucose metabolism of the *hst3*Δ *hst4*Δ cells, which has been similar to those of aged cells, might be a cause to reduce the CLSs.

**Figure 3:**
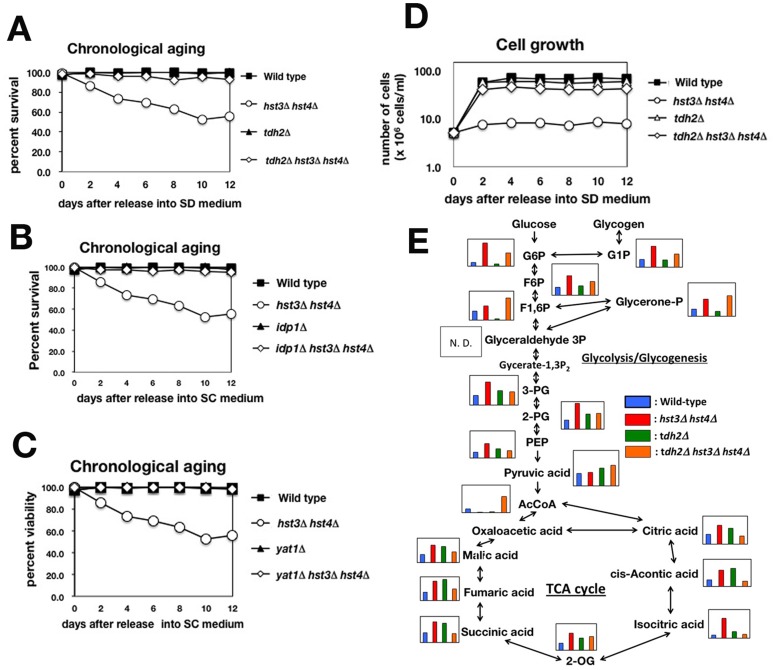
Deletion of the *TDH2* gene restores both CLS and vegetative growth and reduces the metabolic intermediates of glucose metabolism in *hst3*Δ *hst4*Δ cells. (A) The CLSs of wild type, *hst3*Δ *hst4*Δ, *tdh2*Δ and *tdh2*Δ *hst3*Δ *hst4*Δ cells. (B) The CLSs of wild type, *hst3*Δ *hst4*Δ, *tdh2*Δ and *tdh2*Δ *hst3*Δ *hst4*Δ cells. (C) The CLSs of wild type, *hst3*Δ *hst4*Δ, *tdh2*Δ and *tdh2*Δ *hst3*Δ *hst4*Δ cells. (D) Growth curves of wild type, *hst3*Δ *hst4*Δ, *tdh2*Δ and *hst3*Δ *hst4*Δ *tdh2*Δ cells in SC medium. (E) The metabolic profiling data of central carbon metabolism in wild type, *hst3*Δ *hst4*Δ, *tdh2*Δ and *hst3*Δ *hst4*Δ *tdh2*Δ cells were mapped onto known pathways. Each graph was derived from the calculated amount of metabolic intermediates shown in Table S3.

### Deletion of the *TDH2* Gene Restored both the CLS and Vegetative Growth of *hst3*Δ *hst4*Δ cells by Decreasing the Metabolic Intermediates of Glucose Metabolism

Given that the accumulation of glucose metabolites resulted in a shortened chronological lifespan in *hst3*Δ *hst4*Δ cells, we hypothesized that reducing the accumulation of metabolic intermediates within the *hst3*Δ *hst4*Δ cells could extend this lifespan. One approach to reducing the amount of metabolites is to delete the gene encoding a metabolic enzyme (Fig. S5; gene map of glucose metabolism). We screened a glucose metabolism gene deletion, which was able to restore the CLS of *hst3*Δ *hst4*Δ cells. As shown in Figure 3A, the deletion of the *TDH2* gene, which encodes a yeast GAPDH [53], was able to completely restore the lifespan of *hst3*Δ *hst4*Δ cells. We tested whether *tdh2*Δ deletion could extend the RLS of *hst3*Δ *hst4*Δ cells. *tdh2*Δ could slightly, but significantly on statistics, extended the RLS of *hst3*Δ *hst4*Δ cells (Figs. S1 and S6). Similar to *TDH2*, we identified two other genes, *IDP1* and *YAT1*, whose deletion rescued the CLS of the *hst3*Δ *hst4*Δ cells (Fig. 3B and C). Idp1 is a mitochondrial NADP-specific isocitrate dehydrogenase that catalyzes the oxidation of isocitrate to α-ketoglutarate [54]. Yat1 is a carnitine acetyltransferase that localizes to the outer membrane of the mitochondrion and is involved in the transport of acetyl-CoA from the cytosol into the mitochondrion [55]. The decreasing pH of media reduces the cell viability in CLS assay [45]. We confirmed that any *tdh2*, *idp1* or *yat1* gene deletion did not increase the pH of medium to restore the viability of *hst3*Δ *hst4*Δ cell in CLS assay (Table S4). Because the biochemical analysis of GAPDH has been already established, we mainly analyzed Tdh2 in this study, as described later. Aged cells usually grow poorly even though the surrounding environment is suitable for cell growth. Similar to aged cells, the *hst3*Δ *hst4*Δ cells grew poorly in synthetic complete (SC) medium, which permitted normal growth of wild type cells (Fig. 3D). Interestingly, *tdh2*Δ *hst3*Δ *hst4*Δ cells grew in SC medium as well as wild type cells (Fig. 3D). Additionally, *idp1*Δ *hst3*Δ *hst4*Δ and *yat1*Δ *hst3*Δ *hst4*Δ cells grew as well as wild type cells (Fig. S7A and B). Thus, the deletion of genes involved in glucose metabolism not only restored the CLS of *hst3*Δ *hst4*Δ cells but also the poor growth.

**Figure 4:**
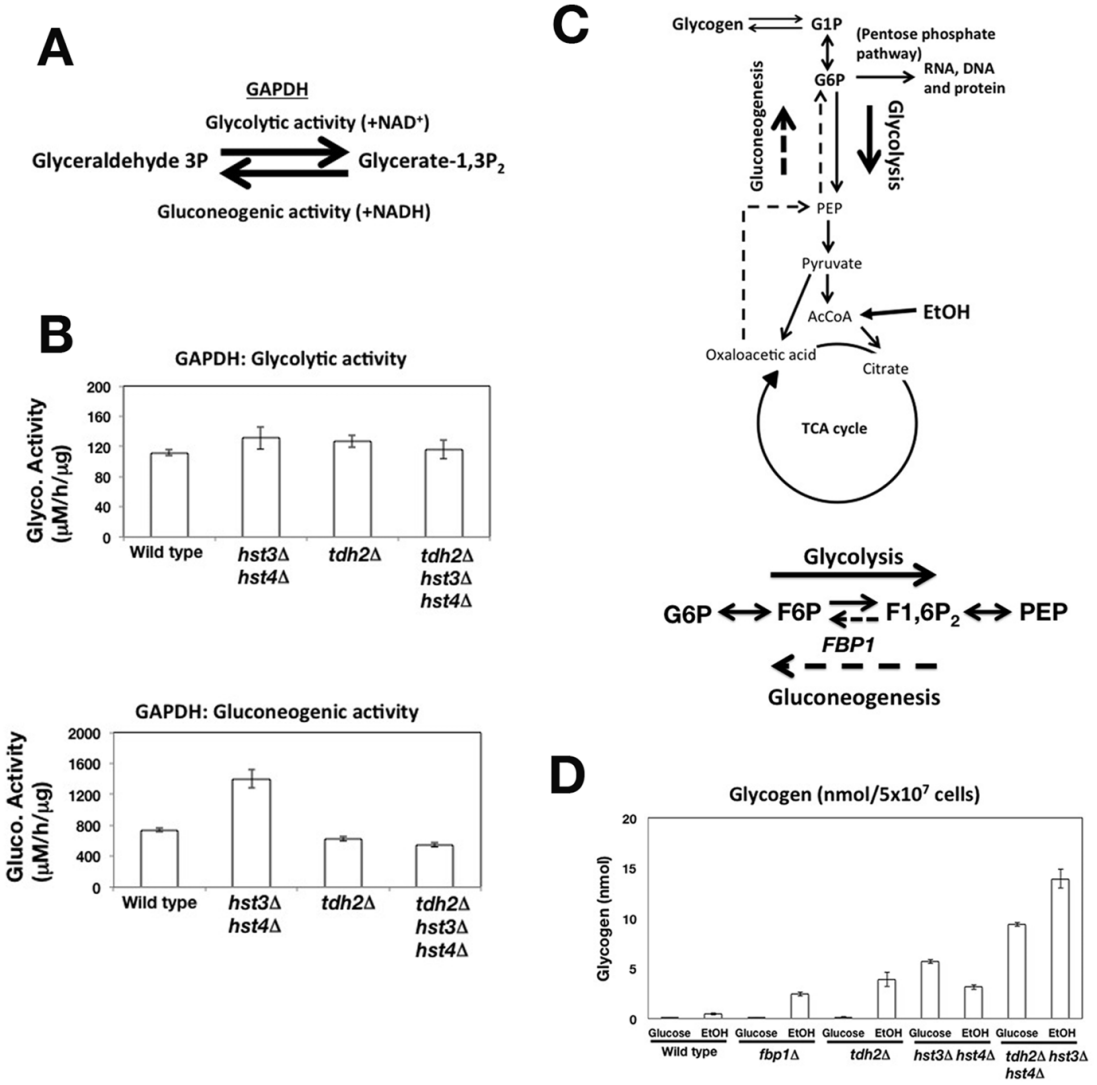
Deletion of the *TDH2* gene can selectively repress the activation of gluconeogenesis. (A) The roles of GAPDH in both glycolysis and gluconeogenesis. (B) Measurement of GAPDH activity. The glycolytic and gluconeogenic activities of GAPDH were monitored using cell lysates prepared from asynchronous cells. (C) A cartoon showing the utilization of ethanol (EtOH) as a non-fermentative carbon source via gluconeogenesis. (D) Glycogen accumulates in gluconeogenesis-deficient cells (*fbp1*Δ) and *tdh2*Δ cells. The amount of glycogen prepared from cells cultured in liquid medium containing either glucose or EtOH was quantified. All graphs show the mean values of measurements performed in triplicate. The error bars denote the standard deviation. Note that certain error bars are too small to be visible.

We analyzed whether the deletion of the *TDH2* gene could reduce the accumulation of metabolic intermediates of glucose metabolism in *hst3*Δ *hst4*Δ cells. Using CE-TOFMS, we compared the profiles of metabolic intermediates among wild type, *hst3*Δ *hst4*Δ, *tdh2*Δ and *tdh2*Δ *hst3*Δ *hst4*Δ cells (Figs. 3E, S8 and Table S3). As shown in Figure 3E, many metabolites that specifically accumulated in *hst3*Δ *hst4*Δ cells (G6P, G1P, and F6P from glycolysis/gluconeogenesis and many metabolites of the TCA cycle) were indeed decreased in *tdh2*Δ *hst3*Δ *hst4*Δ cells. These data suggest that the *tdh2* gene deletion contributed to the decrease in the accumulation of metabolic intermediates of glucose metabolism in *tdh2*Δ *hst3*Δ *hst4*Δ cells.

### Deletion of the *TDH2* Gene Specifically Repressed the Activation of Gluconeogenesis

Next, we investigated whether the *tdh2* gene deletion could specifically repress the gluconeogenesis pathway. We initially examined the influence of the *tdh2* gene deletion on cellular GAPDH activities that play a pivotal role in glycolysis/gluconeogenesis. In the presence of NAD^+^, GAPDH promotes glycolysis to catalyze the conversion of glyceraldehyde-3-phosphate (glyceraldehyde-3P) to glycerate-1,3-bisphosphate (glycerate-1,3P_2_) (Fig. 4A: glycolytic activity). In the presence of NADH, GAPDH promotes gluconeogenesis to catalyze the conversion of glycerate-1,3P_2_ to glyceraldehyde 3P (Fig. 4A: gluconeogenic activity). We prepared cell extracts from wild type, *tdh2*Δ, *hst3*Δ *hst4*Δ and *tdh2*Δ *hst3*Δ *hst4*Δ cells cultured in medium containing glucose as the carbon source and compared the glycolytic and gluconeogenic activities of GAPDH among the cell extracts. The glycolytic activities of GAPDH were similar in all of the cell extracts (Fig. 4B: glycolytic activity). The gluconeogenesis pathway is usually repressed in the presence of glucose, and therefore the gluconeogenic activity of GAPDH should be low. However, the gluconeogenic activity of GAPDH in the *hst3*Δ *hst4*Δ cell extract was significantly higher than that in the wild type cell extract (Fig. 4B: gluconeogenic activity). Interestingly, the gluconeogenic activity in the *tdh2*Δ *hst3*Δ *hst4*Δ cell extract was approximately the same as that observed in both the wild type and *tdh2*Δ cell extracts (Fig. 4B: gluconeogenic activity). Thus, the gluconeogenic activity of GAPDH was increased in *hst3*Δ *hst4*Δ cells. In addition, the gluconeogenic activity of GAPDH in the *hst3*Δ *hst4*Δ cells was repressed by the deletion of the *TDH2* gene without affecting the glycolytic activity.

We next addressed whether the *tdh2* gene deletion repressed the entire gluconeogenesis pathway in the *hst3*Δ *hst4*Δ cells by suppressing the gluconeogenic activity of GAPDH. Budding yeast cells employ gluconeogenesis to utilize non-fermentable carbon sources such as glycerol or ethanol [56]. Ethanol is metabolized in the TCA cycle, and the hexose phosphates produced by gluconeogenesis are used for the biosynthesis of DNA, RNA and proteins via the pentose phosphate pathway (Fig. 4C: cartoon). Because gluconeogenesis-deficient mutant cells are unable to utilize ethanol as a carbon source, we expected that the mutant cells would respond similarly to glucose starvation in the presence and absence of ethanol. Under glucose starvation, yeast cells rapidly accumulate glycogen, a branched polymeric glucose reserve generated from G1P (Fig. 4C) [57], [58]. Previous research reported that *tdh2*Δ cell grows slower than wild type cell in the presence of ethanol [59], which suggest that the gluconeogenesis pathway does not work well in *tdh2*Δ cell. We analyzed the accumulation of glycogen to determine if the gluconeogenesis pathway was intact or deficient. When wild type cells were cultured in the presence of ethanol, the amount of glycogen that accumulated increased slightly compared to that in the presence of glucose (Fig. 4D: wild type). Cells lacking the *FBP1* gene encoding fructose-1,6-bisphosphatase, which irreversibly converts F1,6P_2_ to F6P, exhibit a defect in gluconeogenesis (Fig. 4C) [60]. As expected, *fbp1*Δ cells accumulated a considerable amount of glycogen in the presence of ethanol (Fig. 4D: *fbp1*Δ). Similar to *fbp1*Δ cells, *tdh2*Δ cells also accumulated glycogen in the presence of ethanol but not in the presence of glucose (Fig. 4D: *tdh2*Δ and *fbp1*Δ). We confirmed that the expression of the *FBP1* gene involved in gluconeogenesis was normally induced in response to non-fermentable carbon sources in the *tdh2*Δ cells (Fig. S9). These findings indicate that the *tdh2*Δ cells were deficient in the activation of gluconeogenesis in a manner similar to that observed in *fbp1*Δ cells, although the gene transcription involved in gluconeogenesis was induced normally.

Next, we examined whether the deletion of *tdh2* could repress gluconeogenesis in the *hst3*Δ *hst4*Δ background. Like *hst3*Δ *hst4*Δ cells, *tdh2*Δ *hst3*Δ *hst4*Δ cells accumulated glycogen even in the presence of glucose (Fig. 4D: *hst3*Δ *hst4*Δ and *tdh2*Δ *hst3*Δ *hst4*Δ). Because several metabolites connected with glycogen synthesis (G6P, G1P and F6P) were accumulated in *tdh2*Δ *hst3*Δ *hst4*Δ cells more than in wild type cells (Fig. 3E), glycogen may be synthesized. Similar to *tdh2*Δ cells, *tdh2*Δ *hst3*Δ *hst4*Δ cells accumulated glycogen in the presence of ethanol, whereas *hst3*Δ *hst4*Δ cells did not (Fig. 4D: *tdh2*Δ, *hst3*Δ *hst4*Δ and *tdh2*Δ *hst3*Δ *hst4*Δ). Taken together, these data suggest that the *tdh2* gene deletion specifically represses gluconeogenesis in *hst3*Δ *hst4*Δ cells.

### *TDH2* Gene Deletion Extends the RLS in a CR-dependent Manner

**Figure 5:**
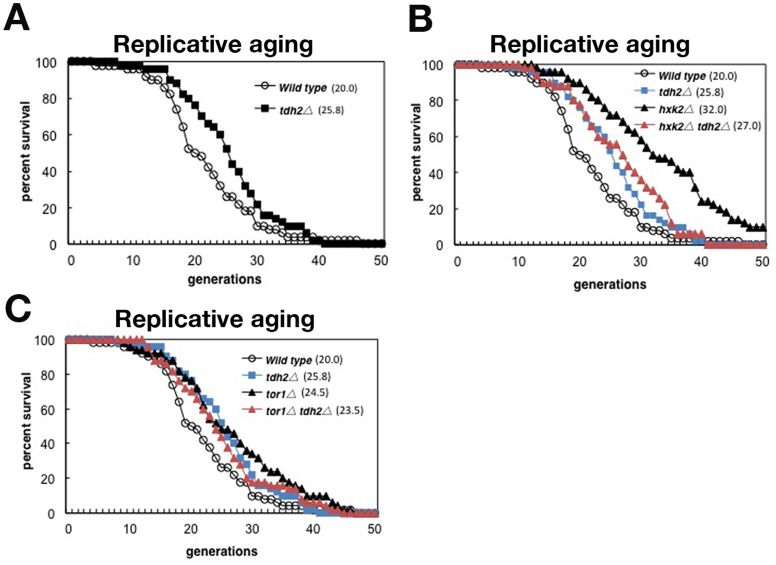
*TDH2* gene deletion extends the RLS in a manner similar to that of the CR pathway. (**A**) The RLSs of wild type and *tdh2*Δ cells. (**B**) The RLSs of wild type, *tdh2*Δ, *hxk2*Δ and *hxk2*Δ *tdh2*Δ cells. (**C**) The RLSs of wild type, *tdh2*Δ, *tor1*Δ and *tor1*Δ *tdh2*Δ cells. The difference between strains was performed on statistical calculations (Fig. S1).

CR reduces the augmentation of gluconeogenesis in aged yeast cells [11]. We examined whether the reduction of gluconeogenesis by the deletion of the *tdh2* gene contributed to the extension of the cellular lifespan in a CR-dependent manner. The RLS of *tdh2*Δ mother cells was significantly extended compared with that of wild type cells (Figs. 5A and S1). To investigate the genetic pathway of *tdh2* gene deletion, we compared the lifespan of the *tdh2* gene deletion in CR-mimicked mutant cells. In one CR model, the deletion of the *HXK2* gene encoding hexokinase reduces the availability of glucose for glycolysis and extends the RLS [61]. In another model of CR, the deletion of *TOR1*, which encodes a phosphatidylinositol kinase (PIK)-related kinase and a subunit of the TORC1 complex, drives cells into a nutrient-starvation mode, extending their RLS [29], [62]. Both *hxk2*Δ and *tor1*Δ single deletion cells significantly had long lifespans (Figs. 5B, C and S1). However, either the RLSs of *hxk2*Δ *tdh2*Δ or *tor1*Δ *tdh2*Δ deletion cell were almost same as that of *tdh2*Δ cell (Figs. 5B, C and S1). Thus, *tdh2* gene deletion can extend the RLS in a CR-dependent manner. These suggest that CR extends RLS by suppressing age-enhanced gluconeogenesis.

## Discussion

Our study suggests that the metabolites in glucose metabolism accumulated in *hst3*Δ *hst4*Δ cells are due to the enhanced gluconeogenesis. However, *hst3*Δ *hst4*Δ *tdh2*Δ cells have still accumulated several metabolites in glucose metabolism (Fig. 3E), even though deletion of *TDH2* gene repressed the activation of gluconeogenesis. To fix DNA damages occurred in the progression of DNA replication forks which are caused by persistent acetylation of histone H3 in K56 in chromatin, *hst3*Δ *hst4*Δ cells grow slowly [24]. Therefore, *hst3*Δ *hst4*Δ cell results in expansion of the cell volume by accumulating various metabolites, which are common to cells mutated in chromosomal DNA replication and cell cycle progression (data not shown). Accumulation of several metabolites in *hst3*Δ *hst4*Δ *tdh2*Δ cells may be derived from a kind of side effect that *hst3*Δ *hst4*Δ *tdh2*Δ cells has expanded to the same cell volume of *hst3*Δ *hst4*Δ cells (data not shown).

Why the gluconeogenesis pathway is activated in *hst3*Δ *hst4*Δ cells is still unclear. Many metabolic enzymes in glucose metabolism, the TCA cycle, the urea cycle, and fatty acid metabolism are acetylated in human liver tissue [63]. In *Salmonella*, metabolic enzymes in glucose metabolism are acetylated extensively and differentially in response to different carbon sources, and the relative activities of key enzymes controlling the balance between glycolysis and gluconeogenesis are particularly regulated by acetylation [42]. Persistent acetylation of metabolic enzymes that should be deacetylated by Hst3 and Hst4 may lead to a defect in the control of the flux between glycolysis and gluconeogenesis. Target proteins of Hst3 and Hst4 that control glucose metabolism and chronological lifespan should be identified in future studies.

In this study, we isolated Idp1 and Yat1 together with Tdh2, which restored the CLS and poor growth of *hst3*Δ *hst4*Δ cells when deleted. It is unclear if either Idp1 or Yat1 can suppress augmented gluconeogenesis in the same manner as Tdh2, although a less direct mechanism may be possible. For example, the deletion of either the Idp1 or Yat1 gene has been proposed to reduce the amount of metabolites to be supplied in glycogen synthesis via gluconeogenesis, which would effectively lead to a reduction in gluconeogenesis. The roles of Idp1 and Yat1 in gluconeogenesis remain to be elucidated.

We also demonstrated that the *tdh2* gene deletion was able to restore the RLS of *hst3*Δ *hst4*Δ cells on statistics, but the effect was marginal (Figs. S1 and S6). Because the short RLS of the *hst3*Δ *hst4*Δ cells is due to the genomic instability [36], the reduction in gluconeogenesis mediated by the *tdh2* gene deletion may not be sufficient to restore the shortened RLS caused by the genomic instability.

It will be interesting to extend our findings to other species, particularly multicellular organisms. In *C. elegans*, a null allele of *Clk-1,* which encodes a protein with homology to an activator of gluconeogenesis in budding yeast (Cat5p), leads to lifespan extension [64]. This finding is consistent with the correlation between reduced gluconeogenesis and increased lifespan observed in yeast ([11] and our study). CR extends lifespan in many species and has been shown to ameliorate many age-associated disorders, such as diabetes and cancer [17]. Testing whether the inactivation of a metabolic enzyme suppresses augmented gluconeogenesis instead of CR would be valuable, and the results could be applied to extend lifespan and ameliorate age-associated disorders.

## Supporting Information

Figure S1 P-value matrices for each figure (Fig. 1A, 5 and S6). Each matrix contains the Wilcoxon Rank-Sum p-value for a 2-tailed test in which the lifespan data for the strain in the corresponding column. Significant p-value (p<0.05) is colored yellow. P-values were calculated using ystat2008 software.
(TIFF)

Figure S2 ROS accumulate specifically in *hst3*Δ *hst4*Δ cells. The number of DHE-positive cells was counted by fluorescence microscopy, and the distribution of DHE-positive cells was calculated (n = 100).
(TIF)

Figure S3 *hst3*Δ *hst4*Δ cells reduce CLS in medium “buffered”. Wild type and *hst3*Δ *hst4*Δ cells cultured in SC medium in the presence “buffered” of 50 mM citrate-phosphate buffer [pH6.0] and 2% glucose. Viability (cultured for 4 days) was measured as colony formation units and calculated as the ratio for the start (0 day). Data represent averages and standard deviations of more than four biological replicas.
(TIFF)

Figure S4 The entire metabolic profiling data set, including central carbon metabolism, for aged cells relative to young cells was mapped onto the network. Each graph was derived from the calculated amount of metabolic intermediates listed in Table S1. The blue and red columns indicate young and aged cells, respectively.
(TIFF)

Figure S5 Map of the central carbon metabolic pathway in budding yeast, based on the information from the *Saccharomyces* genome database (http://www.yeastgenome.org/). The gene(s) involved in each metabolic reaction are shown in red.
(TIF)

Figure S6 The deletion of the *TDH2* gene results in the inability of *hst3*Δ *hst4*Δ cells to extend their RLS. The RLSs of wild type, *tdh2*Δ, *hst3*Δ *hst4*Δ and *hst3*Δ *hst4*Δ *tdh2*Δ cells. The median lifespan is given next to each genotype. The difference between strains was performed on statistical calculations (Figure S1).
(TIFF)

Figure S7 The deletion of either the *IDP1* or *YAT1* gene can restore the growth of *hst3*Δ *hst4*Δ cells. (**A**) The growth curve of wild type, *hst3*Δ *hst4*Δ, *idp1*Δ, and *hst3*Δ *hst4*Δ *idp1*Δ cells in SD medium. (**B**) The growth curve of wild type, *hst3*Δ *hst4*Δ, *yat1*Δ, and *hst3*Δ *hst4*Δ *yat1*Δ cells in SD medium.
(TIF)

Figure S8 The entire metabolic profiling data set, including central carbon metabolism, for *hst3*Δ *hst4*Δ cells relative to wild type cells was mapped onto the network. Each graph was derived from the calculated amount of metabolic intermediates listed in Table 3. Blue column: wild type; red: *hst3*Δ *hst4*Δ; green: *tdh2*Δ; orange: *tdh2*Δ *hst3*Δ *hst4*Δ.
(TIFF)

Figure S9 *FBP1* expression is induced in both wild type and *tdh2*Δ cells in response to ethanol (EtOH). RNA was prepared from cells treated with either glucose or EtOH as a carbon source. Reverse transcription polymerase chain reaction (RT-PCR) was employed to measure the ratio of *FBP1* mRNA to *ACT1* mRNA. All graphs represent the mean of triplicate experiments. The error bars denote the standard deviation.
(TIFF)

Method S1 (DOCX)

Table S1 The metabolic intermediates in aged and young cells. The signal peaks of the metabolic intermediates were converted and normalized to the resultant relative area values, including glucose metabolism intermediates, TCA cycle intermediates, and amino acids. The ratio of the resultant relative area values for aged and young cells is also given.
(XLSX)

Table S2 The metabolic intermediates in wild type and *hst3*Δ *hst4*Δ cells. The calculated amounts of the metabolic intermediates of central carbon metabolism are given. The ratio of the resultant relative area values for wild type and *hst3*Δ *hst4*Δ cells is also given.
(XLSX)

Table S3 The metabolic intermediates among wild type, *tdh2*Δ, *hst3*Δ *hst4*Δ and *tdh2*Δ *hst3*Δ *hst4*Δ cells. The signal peaks of the metabolic intermediates were converted and normalized to the resultant relative area values, including glucose metabolism intermediates, TCA cycle intermediates, and amino acids. The ratio of the resultant relative area values for aged and young cells is also given.
(XLSX)

Table S4 The pH of media of wild type, *hst3*Δ *hst4*Δ, *tdh2*Δ, *yat1*Δ, *idp1*Δ, *hst3*Δ *hst4*Δ *tdh2*Δ, *hst3*Δ *hst4*Δ *yat1*Δ *and hst3*Δ *hst4*Δ *idp1*Δ. Cells were cultured in SD medium in the absence “unbuffered” of 50 mM citrate-phosphate buffer. The pH of fresh medium is set as pH6.0. Cells were cultured at 30°C for 8 days, and pH of media was measured.
(TIFF)
