# Supplementary figures and images for: A Reduction in Age-Enhanced Gluconeogenesis Extends Lifespan
Source: PLoS One. 2013 Jan 14;8(1):e54011. doi: 10.1371/journal.pone.0054011 (PMC3544673; doi:10.1371/journal.pone.0054011)

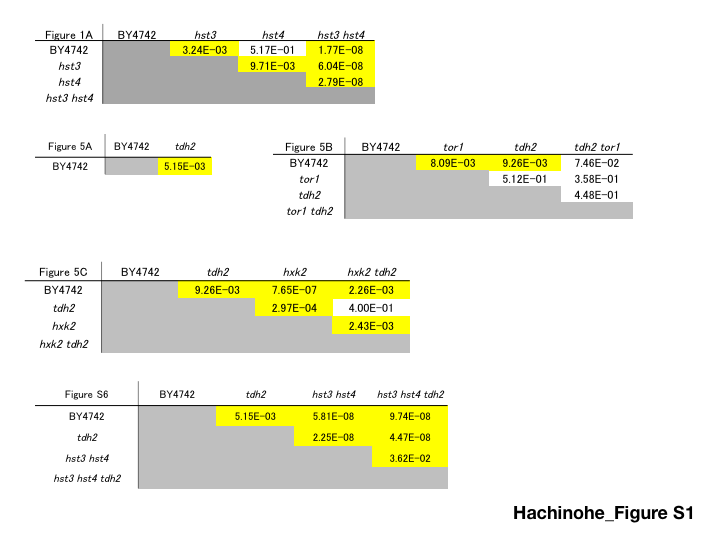

Supplement: Figure S1 — P-value matrices for each figure (Fig. 1A, 5 and S6). Each matrix contains the Wilcoxon Rank-Sum p-value for a 2-tailed test in which the lifespan data for the strain in the corresponding column. Significant p-value (p<0.05) is colored yellow. P-values were calculated using ystat2008 software. (TIFF) [file pone.0054011.s001.tiff]

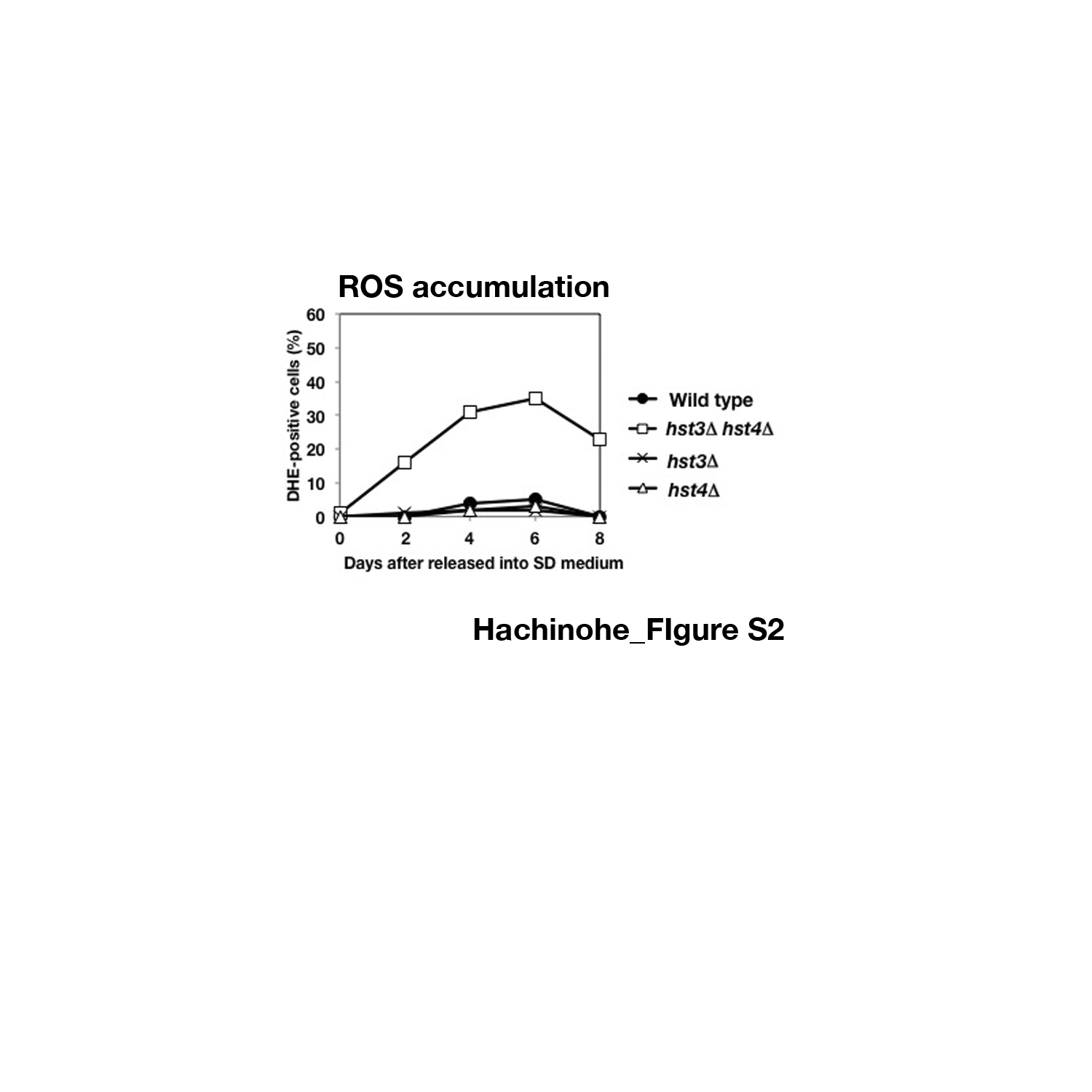

Supplement: Figure S2 — ROS accumulate specifically in hst3Δ hst4Δ cells. The number of DHE-positive cells was counted by fluorescence microscopy, and the distribution of DHE-positive cells was calculated (n = 100). (TIF) [file pone.0054011.s002.tif]

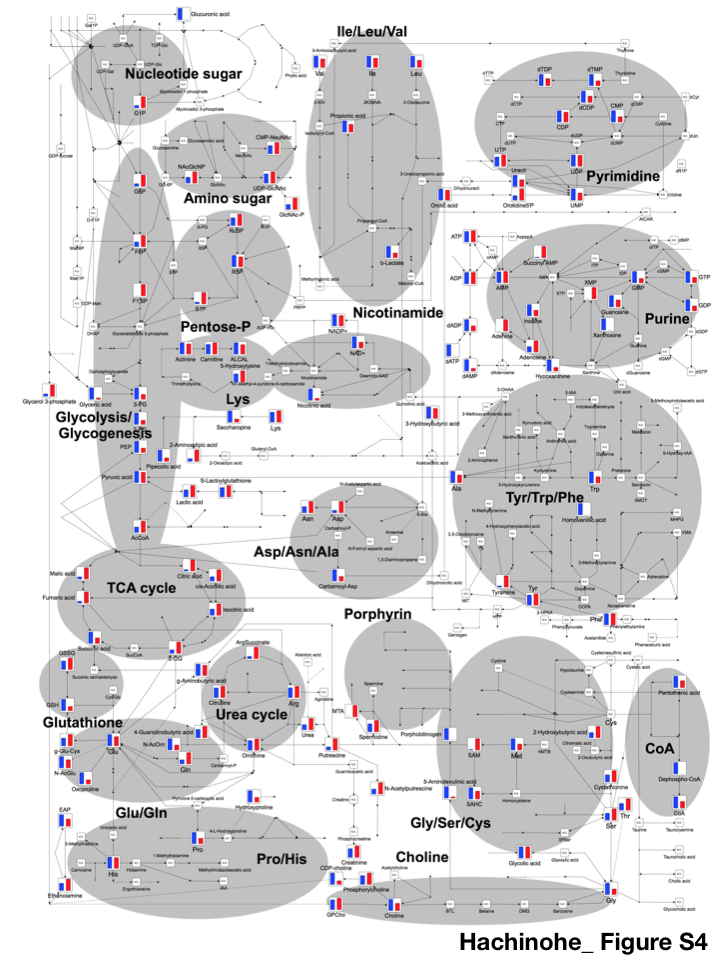

Supplement: Figure S4 — The entire metabolic profiling data set, including central carbon metabolism, for aged cells relative to young cells was mapped onto the network. Each graph was derived from the calculated amount of metabolic intermediates listed in Table S1. The blue and red columns indicate young and aged cells, respectively. (TIFF) [file pone.0054011.s004.tiff]

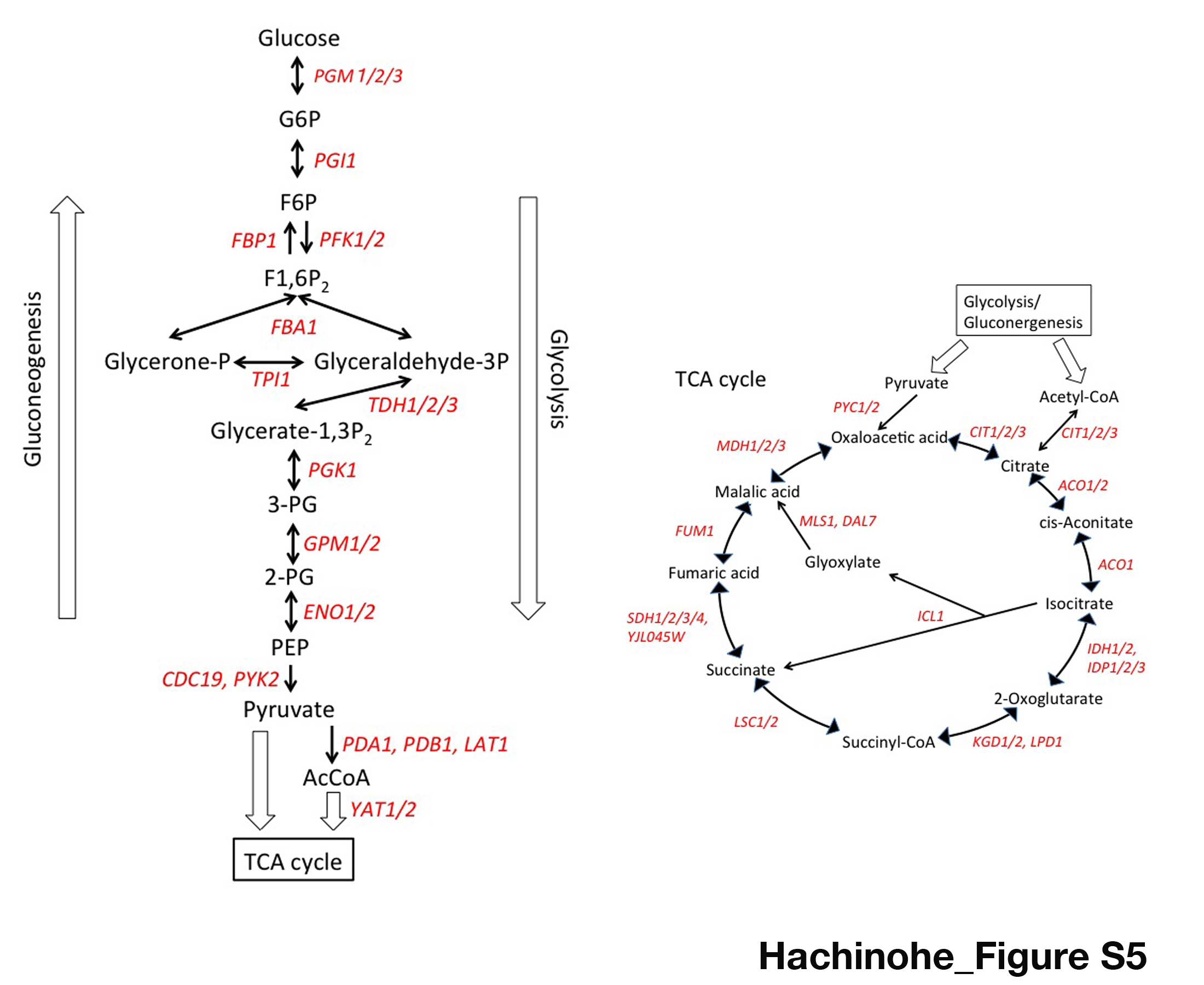

Supplement: Figure S5 — Map of the central carbon metabolic pathway in budding yeast, based on the information from the Saccharomyces genome database (http://www.yeastgenome.org/). The gene(s) involved in each metabolic reaction are shown in red. (TIF) [file pone.0054011.s005.tif]

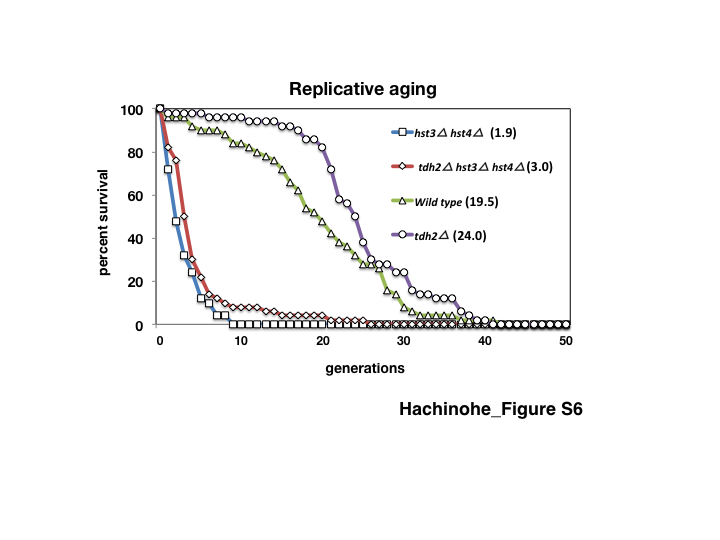

Supplement: Figure S6 — The deletion of the TDH2 gene results in the inability of hst3Δ hst4Δ cells to extend their RLS. The RLSs of wild type, tdh2Δ, hst3Δ hst4Δ and hst3Δ hst4Δ tdh2Δ cells. The median lifespan is given next to each genotype. The difference between strains was performed on statistical calculations (Figure S1). (TIFF) [file pone.0054011.s006.tiff]

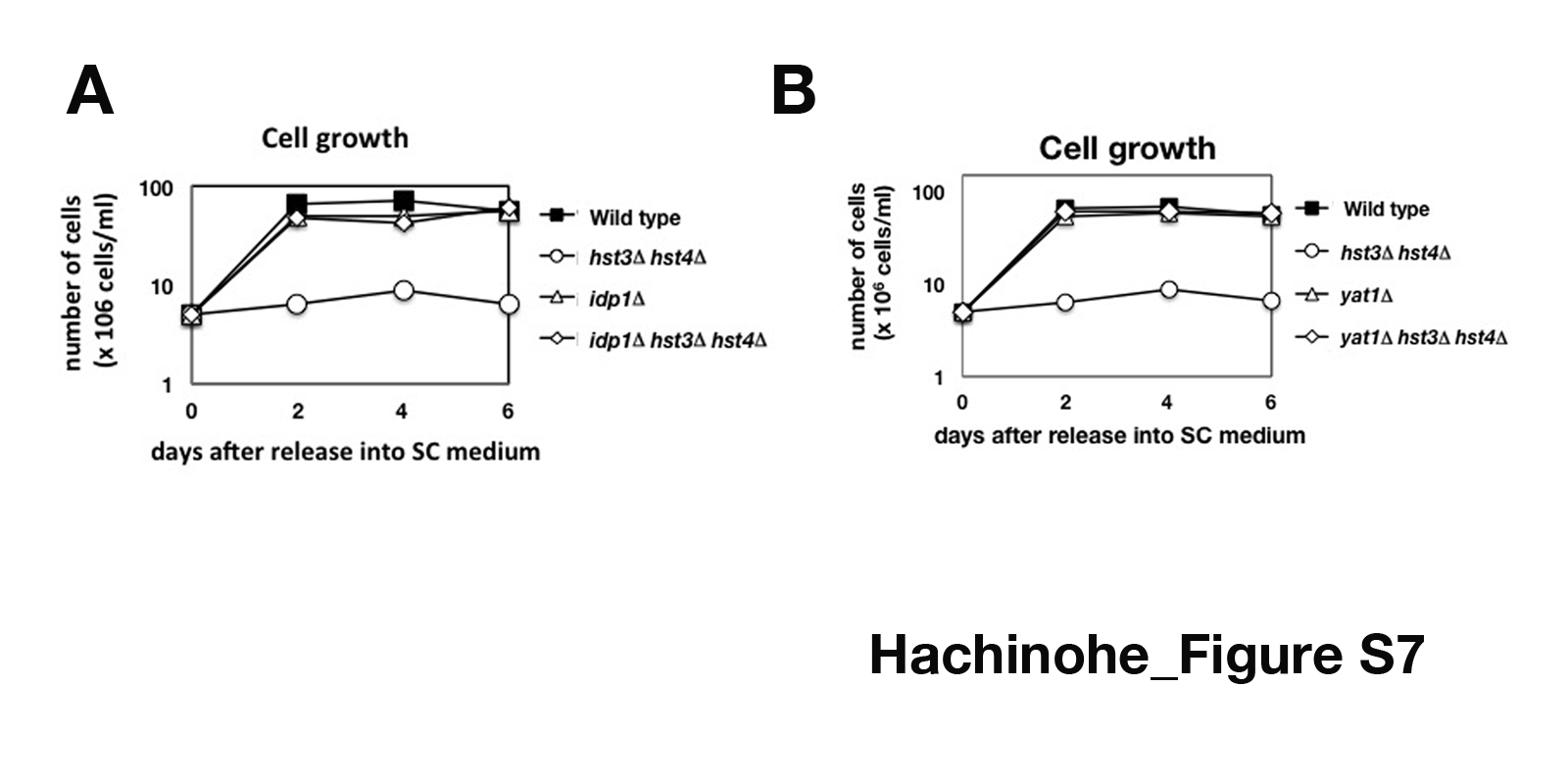

Supplement: Figure S7 — The deletion of either the IDP1 or YAT1 gene can restore the growth of hst3Δ hst4Δ cells. (A) The growth curve of wild type, hst3Δ hst4Δ, idp1Δ, and hst3Δ hst4Δ idp1Δ cells in SD medium. (B) The growth curve of wild type, hst3Δ hst4Δ, yat1Δ, and hst3Δ hst4Δ yat1Δ cells in SD medium. (TIF) [file pone.0054011.s007.tif]

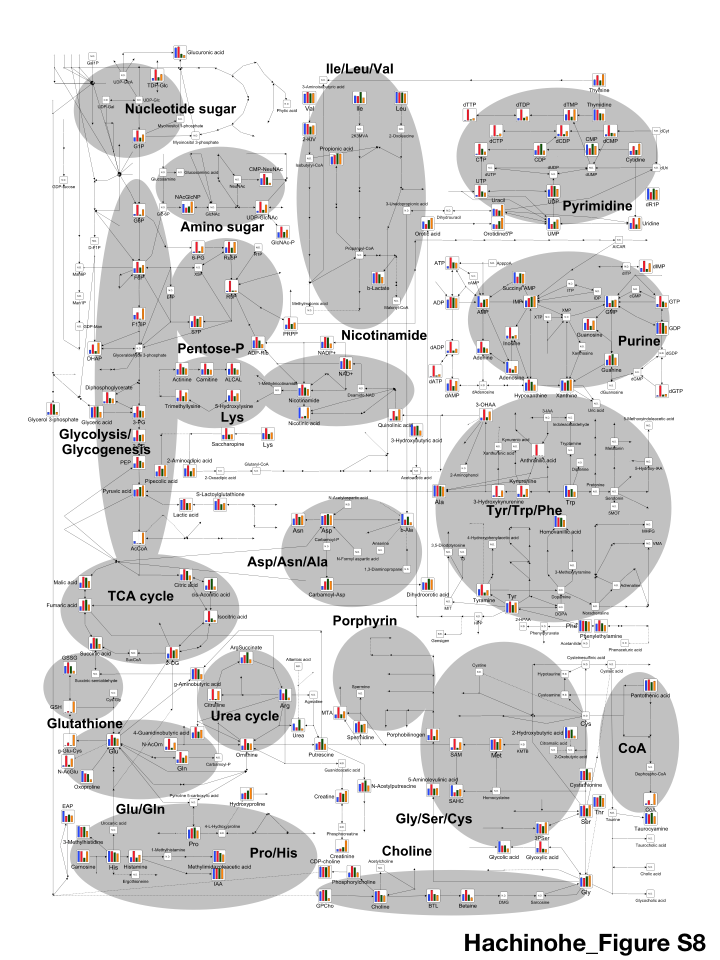

Supplement: Figure S8 — The entire metabolic profiling data set, including central carbon metabolism, for hst3Δ hst4Δ cells relative to wild type cells was mapped onto the network. Each graph was derived from the calculated amount of metabolic intermediates listed in Table 3. Blue column: wild type; red: hst3Δ hst4Δ; green: tdh2Δ; orange: tdh2Δ hst3Δ hst4Δ. (TIFF) [file pone.0054011.s008.tiff]
