# Supplementary material for: A Reduction in Age-Enhanced Gluconeogenesis Extends Lifespan
Source: PLoS One. 2013 Jan 14;8(1):e54011. doi: 10.1371/journal.pone.0054011 (PMC3544673; doi:10.1371/journal.pone.0054011)
Supplement: Method S1 — (DOCX) [file pone.0054011.s010.docx]

**Supporting method S1**

**Strains and media**

The parental *Saccharomyces cerevisiae* strain used in this study was BY4742. The genotypes of the strains used are listed. Unless otherwise stated, all yeast strains were derived from the parent strain for the haploid yeast open reading frame deletion collection [1] available commercially (Open Biosystems Co., USA) and BY4742. To construct gene deletions, we adapted a polymerase chain reaction (PCR)-based procedure to disrupt the target gene in the yeast chromosome [2]. All constructs were confirmed by PCR amplification. Double or triple gene deletion strains were constructed by crossing and sporulation. Because the *hst3*∆ *hst4*∆ double deletion strain harbors the PHM286 *URA3* plasmid, which encodes wild type *HST3* and prevents spontaneous DNA damage and genomic instability, we counter-selected cells for the loss of the PHM286 plasmid by the addition of 5-fluoroorotic acid (5-FOA) prior to usage.

We routinely employed YPD liquid medium (2% Bacto-peptone [BD Difco, NJ, USA], 1% yeast extract, 2% glucose). The YPE liquid medium contained YPD medium and ethanol at a final concentration of 2% instead of glucose. The solid YPD medium contained agar at a final concentration of 2%.

Yeast strains used in this study (BY4742 genetic background)

| **Strain** | **Genotype** | **Reference** | |
| --- | --- | --- | --- |
| BY4742 | *MAT*α *his3*Δ *leu2*Δ*1 met15*Δ*0 ura3*Δ*0* | | [1] |
| *tdh2*Δ | *tdh2*Δ::*kanMX4+* | | [1] |
| *asf1*Δ | *asf1*Δ::*kanMX4+* | | [1] |
| *rtt109*Δ | *rtt109*Δ::*kanMX4+* | | [1] |
| *idp1*Δ | *idp1*Δ::*kanMX4+* | | [1] |
| *tor1*Δ | *tor1*Δ::*kanMX4+* | | [1] |
| *hxk2*Δ | *hxk2*Δ::*kanMX4+* | | [1] |
| *fbp1*Δ | *fbp1*Δ::*his5+* | | This study |
| *yat1*Δ | *yat1*Δ::*kanMX4+* | | [1] |
| HMY368 | *hst3*Δ::*his5+ hst4*Δ*::hph*+[PHM286] | | [3] |
| HMY906 | *MAT*α* idp1*Δ::*kanMX4+hst3*Δ::*his5+ hst4*Δ::*hph+* [PHM286] | | This study |
| HMY910 | *MAT*α *tdh2*Δ::*kanMX4+ hst3*Δ::*his5+ hst4*Δ::*hph*+[PHM286] | | This study |
| HMY918 | *MAT*α* yat1*Δ::*kanMX4+ hst3*Δ::*his5+ hst4*Δ::*hph+* [PHM286] | | This study |
| HMY1136 | *MAT*α* asf1*Δ::*kanMX4+ hst3*Δ::*his5+ hst4*Δ::*hph+* [PHM286] | | This study |
| HMY1141 | *MAT*α* rtt109*Δ::*kanMX4+hst3*Δ*::his5+ hst4*Δ::*hph+* [PHM286] | | This study |
| HMY1236 | *MAT*α *hxk2*Δ::*kanMX4+tdh2*Δ::*his5+* | | This study |
| HMY1256 | *MAT*α *tor1*Δ::*kanMX4+tdh2*Δ::*his5+* | | This study |

**Growth rate assay in liquid culture**

Cells were cultured overnight in YPD at 25°C. The cells (5x10^6^ cells/ml) were suspended in SC medium (0.5% glucose) and cultured at 25°C without shaking. The cell number was determined every 24 h with a Z-1 Coulter Counter (Beckman-Coulter. Co., USA). At least three replicates were analyzed for each strain.

**Reactive oxygen species (ROS) detection**

The protocol was modified as previously described [4]. The cells (5x10^7^) were washed with PBS (pH 7.3) and resuspended in 100 μl of PBS containing 5 µg/ml dihydroethidium (DHE). After incubation at room temperature for 5 min, the cells were harvested and suspended in 100 µl of PBS (pH 7.3). DHE-positive cells were viewed under rhodamine fluorescence using a Leica CTR6500 microscope with LAS AF software. The ratio was calculated from the number of DHE-positive cells among the total number of cells (n=100).

**Metabolome analysis**

**Preparation of old cells for metabolome analysis**

Old cells were isolated by following a modified biotin-streptavidin magnetic sorting method reported previously [5,6]. The BY4742 strain was cultured in 50 ml YPDXtra (2% Bacto-peptone, 1% yeast extract, 3% glucose) liquid medium at 30°C for at least 6 h to an OD_600_ of 0.7. The cells were harvested, washed twice in phosphate-buffered saline (PBS) (pH 7.3) and resuspended in 1 ml of PBS (pH 7.3). Sulfo-NHS-LC-biotin (Pierce, IL, USA) (8–10 mg) was added to the cells, which were then incubated at room temperature with gentle shaking for 15 min. The cells were washed 4 times in 1 ml PBS and resuspended in 1 ml PBS (pH 7.3). The cells (1 × 10^8^) were added to 1000 ml of YPDXtra and grown at 30°C for 13 h with shaking (OD_600_ should not exceed 1.0) (1st sorting). The cells were harvested by centrifugation, washed twice with cold PBS and suspended in 0.5 ml of PBS (pH 7.3).

The biotin-labeled cells were sorted with streptavidin-coated magnetic beads as described previously [7]. The cell suspension was incubated with 200 µl of streptavidin-coated magnetic beads (Miltenyi Biotec GmbH, Germany) for 30 min at RT. The cells were pelleted, washed twice with PBS, resuspended in 8 ml of PBS and then loaded onto a LS MACS column (Miltenyi Biotec GmbH, Germany) that was pre-washed with 2 ml of PBS. The flowthrough fraction was collected as the “Young cells.” The column was washed with 8 ml of PBS, removed from the magnetic field, and eluted with 8 ml of PBS. For double sorts, 1 × 10^8^ cells were added to 1000 ml of YPDXtra and grown at 30°C for 13 h with shaking (OD_600_ should not exceed 1.0). The cells were pelleted, washed twice with PBS, resuspended in 8 ml of PBS and then loaded onto a LS MACS column. The column was washed with 8 ml of PBS, removed from the magnetic field, and eluted with 8 ml of PBS. The OD of the eluent was determined. These harvested cells were collected as the “Aged cells.” The cells (OD_600_= less than 10) were routinely employed for CE-TOFMS analyses.

**Calcofluor staining**

Cells (1x10^6^) were resuspended in 500 µl PBS and fixed with 4% paraformaldehyde for 10 min. The cells were washed three times with PBS, pH 7.3. The number of bud scars was determined by visualization method. Approximately 10^6^ cells were stained in PBS and 10 mg/ml calcofluor white M2R (fluorescent brightener 28) (Invitrogen Co., USA), washed once with 1 ml PBS and viewed under UV fluorescence using a Leica CTR6500 microscope with LAS AF software (Leica Microsystems GMBH, Germany).

**Cell preparation for metabolome analysis**

Unless otherwise noted, cells were cultured overnight in YPD at 25°C. The cells (5x10^6^ cells/ml) were suspended in SC medium (0.5% glucose) and cultured at 30°C with continuous shaking for 3 h. Because the *hst3*Δ *hst4*Δ cells are temperature-sensitive [8], *hst3*Δ *hst4*Δ and *tdh2*Δ *hst3*Δ *hst4*Δ cells were cultured at 25°C. The cells (OD_600_= 20) were routinely employed for CE-TOFMS analyses.

**Measurement of ionic metabolites using a CE-TOFMS system**

The cells were collected by a filtering system (Corning, USA). The filter was washed twice with 10 ml of ultrapure water (LC/MS grade) (Wako, Japan) and then suspended in a dish in 2 ml of methanol containing 10

 µM Internal Standard Solution 1 (Human Metabolome Technologies, Tsuruoka, Japan). The cell suspension (1.6 ml) was transferred to a Falcon tube. Then, 640 µl of Milli-Q water and 1,600 µl of chloroform were added to the samples, which were thoroughly mixed and then centrifuged at 2,300 x *g* for 5 min at 4ºC. The upper aqueous layer (750 µl) was centrifugally filtered through a Millipore 5-kDa cutoff filter to remove proteins. The filtrate was lyophilized and suspended in 25 µl of Milli-Q water.

**Instrumentation**

CE-TOFMS was conducted using an Agilent CE Capillary Electrophoresis System equipped with an Agilent 6210 Time of Flight mass spectrometer, Agilent 1100 isocratic HPLC pump, Agilent G1603A CE-MS adapter kit, and Agilent G1607A CE-ESI-MS sprayer kit (Agilent Technologies, Germany). The system was controlled using Agilent G2201AA ChemStation software version B.03.01 for CE (Agilent Technologies, Germany).

**CE-TOFMS conditions**

Cationic metabolites were analyzed with a fused silica capillary (50

µm i.d.

×

80

cm total length) with Cation Buffer Solution (Human Metabolome Technologies) as the electrolyte. The sample was injected at a pressure of 50

mbar for 10

sec (approximately 10

nl). The applied voltage was set at 27

kV. Electrospray ionization-mass spectrometry (ESI-MS) was conducted in the positive ion mode, and the capillary voltage was set at 4,000

V. The spectrometer was scanned from m/z 50 to 1,000. Other conditions were set as in the cation analysis [9].

Anionic metabolites were analyzed with a fused silica capillary (50

µm i.d.

×

80

cm total length) with Anion Buffer Solution (Human Metabolome Technologies) as the electrolyte. The sample was injected at a pressure of 50 mbar for 25

sec (approximately 25

nl). The applied voltage was set at 30

kV. ESI-MS was conducted in the negative ion mode, and the capillary voltage was set at 3,500

V. The spectrometer was scanned from m/z 50 to 1,000. Other conditions were set as in the anion analysis [10].

**Data analysis**

The raw data obtained by CE-TOFMS were processed with MasterHands software [11]. Signal peaks corresponding to isotopomers of 108 compounds, including glucose metabolism intermediates, TCA cycle intermediates, and amino acids, were extracted. Then, their migration times (MT) were normalized using the migration times of the internal standards. The resultant relative area values were further normalized by the sample amount. The metabolic pathway map was obtained from the public-domain software, VANTED: Visualization and Analysis of Networks containing Experimental Data [12].

**References for Methods**

**1. Winzeler EA, Shoemaker DD, Astromoff A, Liang H, Anderson K, et al. (1999) Functional characterization of the S. cerevisiae genome by gene deletion and parallel analysis. Science 285: 901-906.**

**2. Longtine MS, McKenzie A, 3rd, Demarini DJ, Shah NG, Wach A, et al. (1998) Additional modules for versatile and economical PCR-based gene deletion and modification in Saccharomyces cerevisiae. Yeast 14: 953-961.**

**3. Hachinohe M, Hanaoka F, Masumoto H (2011) Hst3 and Hst4 histone deacetylases regulate replicative lifespan by preventing genome instability in Saccharomyces cerevisiae. Genes Cells 16: 467-477.**

**4. Klinger H, Rinnerthaler M, Lam YT, Laun P, Heeren G, et al. (2010) Quantitation of (a)symmetric inheritance of functional and of oxidatively damaged mitochondrial aconitase in the cell division of old yeast mother cells. Exp Gerontol 45: 533-542.**

**5. Smeal T, Claus J, Kennedy B, Cole F, Guarente L (1996) Loss of transcriptional silencing causes sterility in old mother cells of S. cerevisiae. Cell 84: 633-642.**

**6. Sinclair DA, Guarente L (1997) Extrachromosomal rDNA circles--a cause of aging in yeast. Cell 91: 1033-1042.**

**7. Lindstrom DL, Gottschling DE (2009) The mother enrichment program: a genetic system for facile replicative life span analysis in Saccharomyces cerevisiae. Genetics 183: 413-422, 411SI-413SI.**

**8. Brachmann CB, Sherman JM, Devine SE, Cameron EE, Pillus L, et al. (1995) The SIR2 gene family, conserved from bacteria to humans, functions in silencing, cell cycle progression, and chromosome stability. Genes Dev 9: 2888-2902.**

**9. Soga T, Heiger DN (2000) Amino acid analysis by capillary electrophoresis electrospray ionization mass spectrometry. Anal Chem 72: 1236-1241.**

**10. Soga T, Ishikawa T, Igarashi S, Sugawara K, Kakazu Y, et al. (2007) Analysis of nucleotides by pressure-assisted capillary electrophoresis-mass spectrometry using silanol mask technique. J Chromatogr A 1159: 125-133.**

**11. Sugimoto M, Wong DT, Hirayama A, Soga T, Tomita M (2010) Capillary electrophoresis mass spectrometry-based saliva metabolomics identified oral, breast and pancreatic cancer-specific profiles. Metabolomics 6: 78-95.**

**12. Junker BH, Klukas C, Schreiber F (2006) VANTED: a system for advanced data analysis and visualization in the context of biological networks. BMC Bioinformatics 7: 109.**
